# Game-based inoculation versus graphic-based inoculation to combat misinformation: a randomized controlled trial

**DOI:** 10.1186/s41235-023-00505-x

**Published:** 2023-07-31

**Authors:** Bo Hu, Xing-Da Ju, Huan-Huan Liu, Han-Qian Wu, Chao Bi, Chang Lu

**Affiliations:** 1 School of Psychology, Northeast Normal University, Changchun, 130024 China; 2 Jilin Provincial Key Laboratory of Cognitive Neuroscience and Brain Development, Changchun, China

**Keywords:** Misinformation, Inoculation, Game-based intervention, Graphic-based intervention

## Abstract

Misinformation affects various aspects of people’s lives, such as politics, entertainment, and social interactions. However, effective intervention measures to combat misinformation are lacking. The inoculation theory has become a prevalent measure of misinformation. This study employed inoculation theory and developed an interactive game to help the public counter misinformation. In this game, players take on the role of the misinformation spreader, intending to add more followers to their virtual accounts using different strategies. A total of 180 Chinese participants were randomly assigned to game-based inoculation, graphic-based inoculation, and control groups. The results indicated that both types of inoculation interventions significantly decreased the perceived credibility and sharing intention of misinformation. Game-based inoculation was more effective than graphic-based inoculation in terms of misinformation perceived credibility, and the intervention effects were stable after 2 weeks. The graphic-based inoculation contained the sleeper effect, which interventions required a period of time to take effect. Neither inoculation produced countereffects on perceived credibility and nor sharing intention of accurate information.

**Supplementary Information:**

The online version contains supplementary material available at 10.1186/s41235-023-00505-x.

## Significance statement

Misinformation has become a severe social problem, and helping the public deal with it is a significant focus of psychologists’ research. One effective approach has been to use psychological inoculation to combat misinformation. Still, the comparative effectiveness of different forms of inoculation and their impact on the perception of accurate information needs further investigation. In this study conducted in China, we compared the effects of game-based and graphic-based forms of inoculation and analyzed their impact on the perception of accurate information and misinformation. We found that both forms of inoculation effectively reduced perceived credibility and sharing intention of misinformation, and the effects remained stable for two weeks. The game-based inoculation was more effective in reducing perceived credibility of misinformation than graphic-based inoculation. Neither form of inoculation impacted the perceived credibility and sharing intention of accurate information. These findings demonstrate the effectiveness of inoculation in combating misinformation and suggest that more active inoculation measures should be developed and applied.

## Introduction

Misinformation affects the daily lives of individuals and the functioning of society. During the COVID-19 pandemic, vaccine-related misinformation has reduced the public’s willingness to be vaccinated (Nuwarda et al., 2022), and some patients have adopted incorrect prevention methods such as refusing to wear masks (Aghababaeian et al., 2020) or taking ineffective remedies (Pennycook et al., 2020), such as consuming alcohol. Moreover, misinformation can lead to political polarization (Scheufele & Krause, 2019) and harm democratic institutions (Ecker et al., 2022).

There are various ways to combat misinformation. For example, experts and relevant organizations conduct fact-checks on misinformation (Paynter et al., 2019). Social media platforms have improved their structures and detection systems to reduce the likelihood of misinformation dissemination (Pennycook et al., 2021a, 2021b; Vosoughi et al., 2017). However, the effects of correction often dissipate quickly, and social media cannot block the appearance of misinformation. Therefore, improving individual abilities is a more effective way to address the shortcomings of the current measures. Researchers have effectively increased the public’s resilience to misinformation using online toolkits and media literacy programs (Guess et al., 2020; McGrew, 2020).

Among the methods used to enhance individuals’ ability to cope with misinformation, interventions based on inoculation theory have been widely applied because of their short intervention time and scalability. The inoculation theory suggests that injecting a weakened dose of a virus can activate the production of antibodies, and the same process can be applied in the context of information processing (Ecker et al., 2022). Inoculation interventions have been used to address these problems. The "Bad News" game was designed by Roozenbeek and van der Linden (2019) to enhance resistance to misinformation strategies, Jolley and Douglas (2017) exposed participants to anti-vaccine arguments to improve resistance to anti-vaccine beliefs, and Agley (2021) presented participants with scientific infographics to prevent online misinformation on COVID-19.

With increasing research, inoculation interventions have evolved from primarily focusing on the content of information toward the technique behind the information and also from passive to active inoculation (van der Linden, 2022). This shift could increase the specificity and scalability of interventions. Roozenbeek and van der Linden (2019) applied six techniques to combat misinformation (discredit, conspiracy, trolling, polarization, impersonation, emotion); Basol et al. (2021) applied three techniques to combat COVID-19 related misinformation (fearmongering, fake experts, and conspiracy). Passive inoculation required participants to passively read the rebuttals provided in the inoculation message (Compton & Pfau, 2005). However, during active inoculation, the participants were required to generate their own rebuttals in response to the rebuttals presented in the message. Active inoculation is thought to be more effective than passive inoculation because the "internal" rebuttal used in active inoculation is a more complex cognitive process (Green et al., 2022). Therefore, this process requires the most time and motivation.

Current misinformation interventions are mainly focused on Western countries. Language and cultural differences in the intervention content also result in a lack of evidence regarding the generalizability of the interventions (Kozyreva et al., 2022). This study was conducted in an Eastern country (China), providing further evidence for the globalization of intervention effects. Although some researchers believe that active interventions perform better (Mayer, 2019), there is inadequate evidence comparing the effects of different forms of misinformation interventions (van der Linden, 2022). Exploring the effects of different forms of inoculation can help relevant institutions choose more effective methods based on the actual needs when implementing interventions. With the development of misinformation research, researchers have found that misinformation interventions may damage trust in accurate information (Guess et al., 2020). Interventions can improve the perceived credibility of misinformation by reducing the perceived credibility of accurate information (). When researchers conduct interventions for misinformation, the potential countereffects of the intervention must be considered.

Based on the above questions, this study designed an online game called "Distinguishing Truth from Misinformation" on WeChat, which is one of the largest Chinese social media platforms. This study examined whether a game-based intervention could improve the perceived credibility and sharing intention of misinformation compared to a graphic-based intervention.

For the present study, we tested the following hypotheses:

### H1

Participants in both game-based and graphic-based inoculation conditions will report less perceived credibility than those in the control condition with misinformation.

### H2

Participants in game-based inoculation conditions will report less perceived credibility than those in the graphic-based inoculation with misinformation.

### H3

Two weeks after exposure to the intervention, participants in the game-based and graphic-based inoculation groups will preserve the intervention effect of perceived credibility for misinformation.

### H4

Participants in both the game-based and graphic-based inoculations will not report lower perceived credibility than those in the control condition with accurate information.

### H5

Participants in both the game-based and graphic-based inoculation conditions will report less sharing intention than those in the control condition with misinformation.

### H6

Participants in game-based inoculation conditions will report less sharing intention than those in the graphic-based inoculation with misinformation.

### H7

Two weeks after exposure to the intervention, participants in the game-based and graphic-based inoculation groups will preserve the intervention effect of sharing the intention for misinformation.

### H8

Participants in both game-based and graphic-based inoculations will not report less sharing intention than in the control condition with accurate information.

## Method

### Intervention design

The effectiveness of the “Bad News” game designed by Roozenbeek and van der Linden (2019) has been confirmed in different environments. This intervention takes the main techniques used in the “Bad News” game and adds suggestions from mainstream platforms for responding to misinformation. Finally, players experience eight techniques in the game: filter bubbles, emotion, impersonation, fake experts, conspiracy theories, false evidence, sponsors behind, and social media robots. Details are presented in Table 1.

**Table 1:** Strategies used in the game and the form of presentation

	Strategies	Presentation
Filter bubbles	Social media presents us with the news we want to see and reinforces our original views (Nguyen, 2020)	During the game, participants experience the process of creating filter bubbles by posting social media posts
Emotion	The emotion of information expression is an essential influence on the perception of misinformation (Gabarron et al., 2021)	During the game, participants will understand the impact of emotional content on the public by posting ordinary social posts versus posts with emotions
Impersonation	Posters of information will use names similar to authoritative accounts to mislead the public by exploiting the reputation of their counterparts (Goga et al., 2015)	During the game experience, participants will change their identity and use more authoritative statements to gain attention
Fake expert	Posters may use non-relevant expert statements or false experts to increase credibility (Kuru et al., 2020)	During the game experience, participants increase their attention through false expert testimonials
Conspiracy theory	People have less trust in institutions and traditional authorities (Pummerer, 2022) and therefore are more likely to believe in the existence of a conspiracy	During the game experience, participants learn about the role of conspiracy allegations in shaping public opinion
False evidence	Information that provides evidential content also increases credibility, and evidence-based misinformation is perceived as more accurate than fact-free misinformation (Hameleers, 2022)	During the game experience, participants experience differences in response to textually presented content versus the addition of graphic content and add false evidence to gain attention
Sponsors behind	Many news contents have hidden sponsors behind them (Scott et al., 2019), which can make the sponsor profitable while losing objectivity (Maani et al., 2022)	During the game experience, participants communicate with the sponsor from a first-person perspective and post social media content that benefits the sponsor
Social media robots	Robots populate social media and can create the false impression that a particular viewpoint has gained widespread public support (Zerback et al., 2021)	During the game experience, participants use robots to increase the discussion in their favor, increasing the exposure of their posted content

Based on inoculation theory, the game aims to educate players about the importance and urgency of the misinformation problem and provide them with resistance strategies against future encounters with misinformation (Basol et al., 2021). In this five-minute game, players act as misinformation spreaders to add more followers to their virtual accounts. During the game, various communication strategies were set up as different dialog options that the player triggered to spread misinformation. Players learn about online misinformation techniques and their consequences. For ethical reasons, the intervention refers to Greene et al. (2022) suggestion: Before the game started, players were alert that they would read misinformation; after the game, the misinformation would be corrected. Details about the game can be seen in Additional file 1: Intervention game screenshots.

### Participants

Considering the effect sizes reported in previous inoculation studies (Roozenbeek & van der Linden, 2019), a priori power analysis was conducted with G* power 3.1 using *α* = 0.05, *f* = 0.26 and power of 0.90 with repeated-measures ANOVA (Faul et al., 2009). The minimum sample size required was 117 participants. A total of 180 participants were recruited from 29 provinces in China; the main group comprised university students. The participants had a mean age of 21.24 (SD = 1.98); 43% (77) were male, 57% (103) were female, 51% (92) were from rural areas, and 49% (88) were from urban areas.

### Procedure

The measurement materials for misinformation used in this study follow the recommendations of Pennycook and Binnendyk (). Materials were selected from China’s mainstream fact-checking platform (www.piyao.org.cn). As Weibo is currently the primary source of information in China (Zhu et al., 2020), news materials are edited in the form of Weibo articles. Information providers and interactions were blurred to avoid their influence. Misinformation and accurate information materials mainly focused on health and safety information during the COVID-19 pandemic between 2020 and 2022. Following Ecker et al. (2020) method for selecting misinformation, 30 Weibo users were recruited to evaluate the materials on a five-point Likert scale. Materials with the following assessment results were excluded: familiarity scores > 4, credibility scores < 2 or > 4, and emotional intensity scores < 2 or > 4. Thirty materials were included (15 true and 15 false responses).

**Fig. 1:**
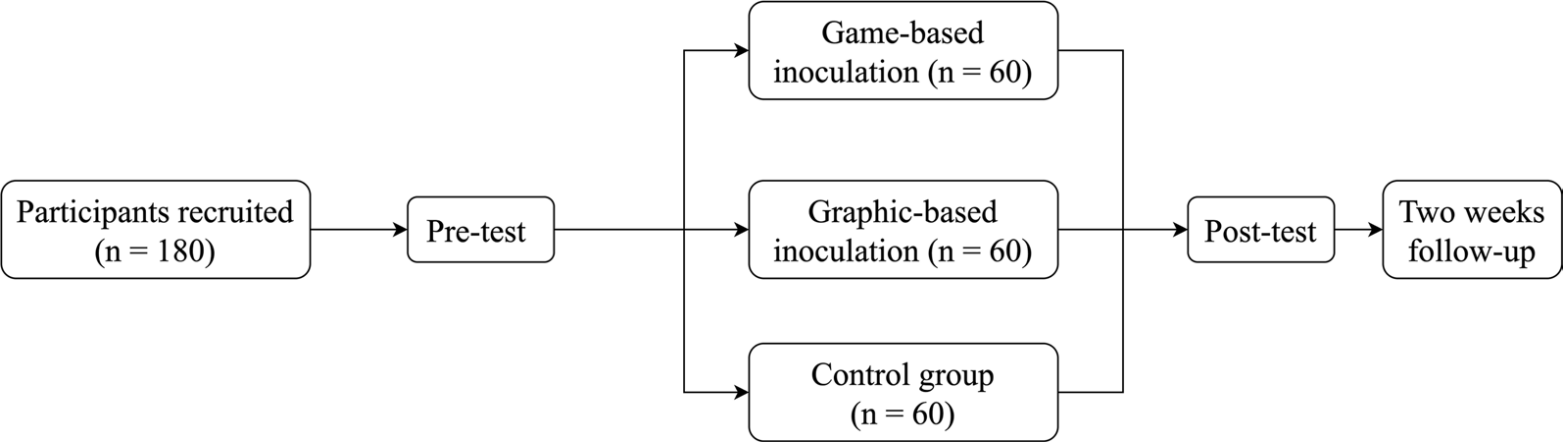
Intervention design flow chart

As shown in Fig. 1, 180 participants were randomly assigned to the game-based inoculation group (*n* = 60), graphic-based inoculation group (*n* = 60), or control group (*n* = 60). The game-based inoculation group played the game designed for this study. The graphic-based inoculation group received the same techniques using graphic materials, simultaneously, as the game-based inoculation. The control group played Tetris simultaneously with the other two groups.

Before the intervention, participants measured the covariates (demographic, media literacy, and cognitive ability). Perceived credibility and sharing intention were assessed at pre-test, post-test, and 2-weeks follow-up. Participants received 10 posts in the form of Weibo (five true and five false) and evaluated the materials using a five-point Likert scale at each stage. After reading each post, each participant was asked, “Is the post above accurate?” (1 = totally not accurate, 5 = very accurate); “Would you consider sharing this post online?” (1 = totally unwilling to share; 5 = very willing to share). Different misinformation materials were presented to participants at different measurement stages. Participants received approximately 1 USD after completing the intervention. Details about the measurement materials can be seen in Additional file 2: Measurement materials.

### Measurement

#### Dependent variables

*Perceived credibility*: For each post, the perceived credibility was based on the average rating on a five-point Likert scale (1 = totally not accurate, 5 = very accurate). A higher perceived credibility score indicated a higher level of belief in the post.

*Sharing intention*: For each post, the sharing intention was evaluated using an average rating on a five-point Likert scale (1 = totally unwilling to share, 5 = very willing to share). A higher sharing intention score indicates that people are more likely to share their posts.

#### Covariates

*Media literacy:* Media literacy is a primary factor that influences individuals’ perceptions of misinformation (Su et al., 2022). This study used the media literacy scale developed by Jones-Jang et al. (2021), which consists of four questions. Sample items include: “I would follow the news using multiple media sources,” and “I would contact with news organizations to show my reaction and tell my criticism.” The Cronbach α for media literacy was 0.915.

*Cognitive ability:* Cognitive ability also influences the susceptibility to misinformation. We used the cognitive ability testing method proposed by Pennycook et al. (2020). The six test questions were designed to elicit automatic and intuitive responses. The score was the number of correct answers given by the participants. The Cronbach α for cognitive ability was 0.902.

## Result

The ANOVA test was conducted on the pre-test questionnaire. There were no significant differences among the three groups in terms of misinformation perceived credibility, *F* (2,177) = 1.584, *p* = 0.208; misinformation sharing intention, *F* (2,177) = 1.421, *p* = 0.244; accurate information perceived credibility, *F* (2,177) = 0.904, *p* = 0.407; and accurate information sharing intention, *F* (2,177) = 0.344, *p* = 0.709. The differences in the demographic variables between the groups, as shown in Table 2, were also not statistically significant.

**Table 2:** Differences in demographic variables among groups (*n* = 180)

Variable	Game-based inoculation	Graphic-based inoculation	Control group	Difference test	*p*
*Gender*					
Male	34	35	26	*X*^*2*^ = 3.254	*p* = .196
Female	26	25	34		
Age	21.27 ± 1.84	21.05 ± 1.98	21.23 ± 2.13	F = 0.207	*p* = .813
Media literacy	3.68 ± 0.46	3.82 ± 0.57	3.80 ± 0.60	F = 1.235	*p* = .293
Cognitive ability	3.51 ± 1.57	3.08 ± 1.57	3.31 ± 1.53	F = 1.166	*p* = .314
*Region*					
Urban	36	29	27	*X*^*2*^ = 6.016	*p* = .416
Countryside	24	31	33		

ANOVA reports the mean and standard deviation (M ± SD) of each group of data, and chi-square analysis reports the number of groups (*n*)

### Misinformation perceived credibility

**Fig. 2:**
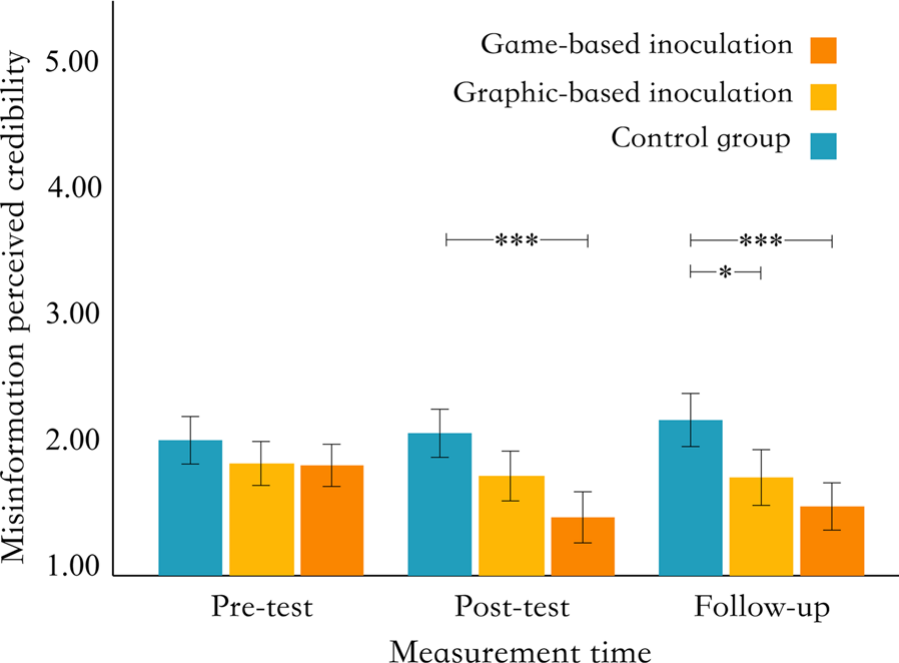
Distribution of misinformation perceived credibility. *Note* * = *p* < .05, ** = *p* < .01, *** = *p* < .001

To test hypotheses H1, H2, and H3, the present study used a one-factor repeated-measures ANOVA to examine the differences in the perceived credibility of misinformation among different groups at different measurement times. The different intervention forms (game-based, graphic-based, and control groups) were the between-subject factors, and the measurement time (pre-test, post-test, and follow-up) was the within-subject factors. The scores for perceived credibility for different measurement times and intervention forms are shown in Fig. 2.

The result showed a significant interaction between intervention forms and measurement time, *F* (4, 354) = 2.65, *p* = 0.033, *η*^*2*^ = 0.014. The main effect of intervention forms was significant, *F* (2, 177) = 15.63, *p* < 0.001, *η*^*2*^ = 0.074, and the main effect of measurement time was not significant,* F* (2, 354) = 2.29, *p* = 0.103, *η*^*2*^ = 0.006. The results of Tukey’s HSD post-hoc tests showed that, under the post-test condition, the misinformation perceived credibility score of the game-based group was significantly lower than that of the control group (*M*_diff_ = − 0.59, *p*_tukey_ < 0.001, *d* = − 0.89, 95% CI [− 1.49, − 0.28]; large effect). The misinformation perceived credibility score of the graphic-based group was not significantly different from that of the control group (*M*_diff_ = − 0.30, *p*_tukey_ = 0.248, *d* = − 0.45, 95% CI [− 1.04, 0.14]; small to intermediate effect). The misinformation perceived credibility score of the game-based group was not significantly different from that of the graphic-based group (*M*_diff_ = − 0.30, *p*_tukey_ = 0.248, *d* = − 0.45, 95% CI [− 1.04, 0.14]; small to intermediate effect).

During the follow-up measurement after 2 weeks, the misinformation perceived credibility score of the game-based group was significantly lower than that of the control group (*M*_diff_ = − 0.61, *p*_tukey_ < 0.001, *d* = − 0.91, 95% CI [− 1.52, − 0.31]; intermediate effect). The graphic-based group was significantly lower than the control group (*M*_diff_ = − 0.40, *p*_tukey_ = 0.026, *d* = − 0.61, 95% CI [− 1.20, − 0.01]; intermediate effect).

### Accurate information perceived credibility

**Fig. 3:**
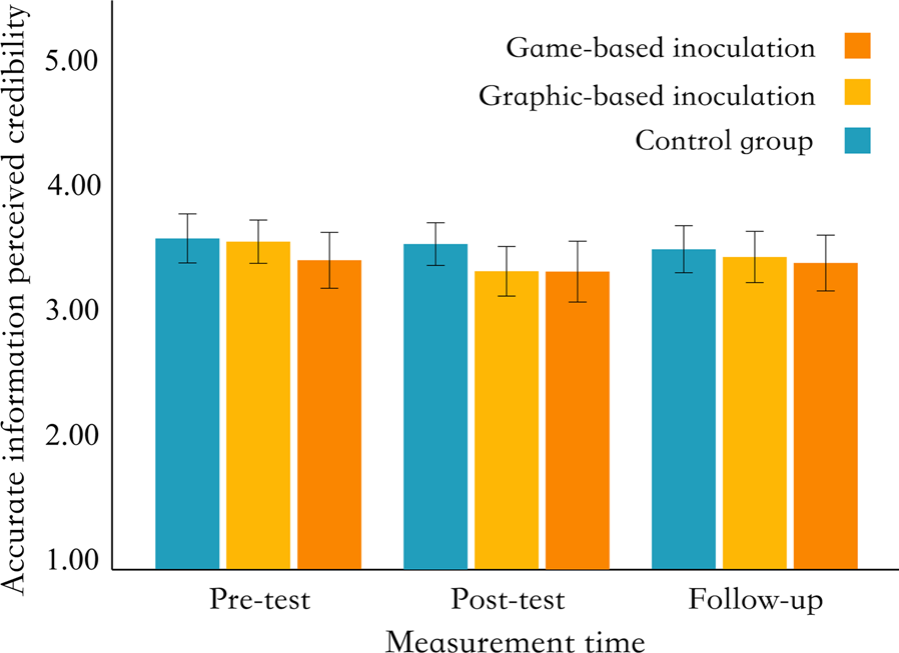
Distribution of accurate information perceived credibility

To test hypothesis H4, a one-factor repeated-measures ANOVA was conducted to examine the differences in the accurate information perceived credibility among different groups, at different measurement times. We did not observe significant effects of the interaction between intervention forms and measurement time (*F* (4, 354) = 0.40, *p* = 0.807, *η*^*2*^ = 0.002), the main effect of intervention forms (*F* (2, 177) = 1.46, *p* = 0.235, *η*^*2*^ = 0.008), and the main effect of measurement time (*F* (2, 354) = 1.41, *p* = 0.246, *η*^*2*^ = 0.004). That means no significant differences in perceived credibility for accurate information between different intervention groups at different measurement times (Fig. 3).

### Misinformation sharing intention

To test hypotheses H5, H6, and H7, a one-factor repeated-measures ANOVA was conducted to examine the differences in misinformation sharing intention among different groups at different measurement times.

**Fig. 4:**
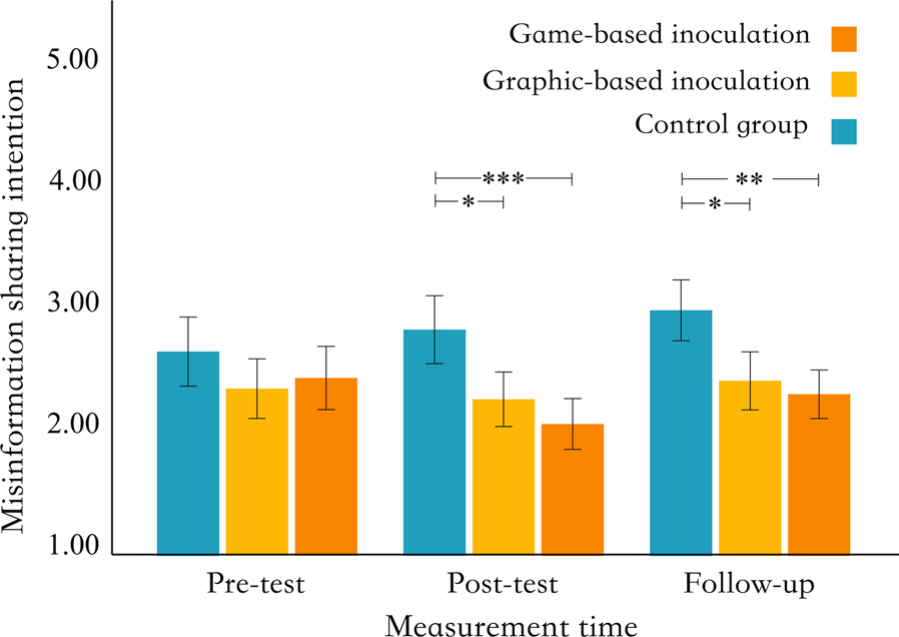
Distribution of misinformation sharing intention. *Note* * = *p* < .05, ** = *p* < .01, *** = *p* < .001

The result showed a significant interaction between intervention forms and measurement time, *F* (4, 354) = 2.47, *p* = 0.044, *η*^*2*^ = 0.011. The main effect of intervention forms was significant, *F* (2, 177) = 10.49, *p* < 0.001, *η*^*2*^ = 0.064, and the main effect of measurement time was not significant,* F* (2, 354) = 2.81, *p* = 0.062, *η*^*2*^ = 0.006. The scores for sharing intention at different measurement times and intervention forms are shown in Fig. 4. Post-hoc Tukey HSD tests revealed that, under the post-test condition, the misinformation sharing intention scores of the game-based group were significantly lower than those of the control group (*M*_diff_ = − 0.68, *p*_tukey_ < 0.001, *d* = − 0.82, 95% CI [− 1.42, − 0.21]; large effect). The graphic-based group was significantly lower than that of the control group (*M*_diff_ = − 0.50, *p*_tukey_ = 0.028, *d* = − 0.60, 95% CI [− 1.20, − 0.01]; intermediate effect). There was no significant difference between the game-based and graphic-based groups (*M*_diff_ = − 0.17, *p*_tukey_ = 0.963, *d* = − 0.21, 95% CI [− 0.80, 0.38]; small effect).

At the 2-weeks follow-up, the scores for misinformation sharing intention in the game-based group remained significantly lower than in the control group (*M*_diff_ = − 0.60, *p*_tukey_ = 0.003, *d* = − 0.73, 95% CI [− 1.33, − 0.13]; intermediate to large effect). The graphic-based group remained significantly lower than the control group (*M*_diff_ = − 0.51, *p*_tukey_ = 0.025, *d* = − 0.61, 95% CI [− 1.21, − 0.02]; intermediate effect).

### Accurate information sharing intention

**Fig. 5:**
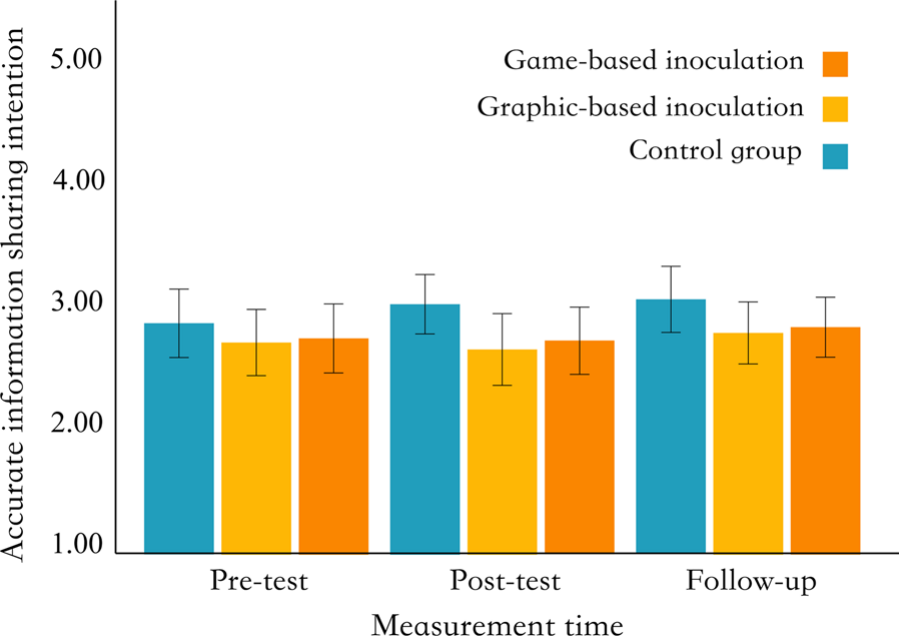
Distribution of accurate information sharing intention

To test hypothesis H8, a one-factor repeated-measures ANOVA was conducted to examine the differences in the accurate information sharing intention among different groups at different measurement times. We did not observe significant effects of the interaction between intervention forms and measurement time (*F* (4, 354) = 0.33, *p* = 0.860, *η*^*2*^ = 0.001), the main effect of intervention forms (*F* (2, 177) = 1.75, *p* = 0.177, *η*^*2*^ = 0.012), and the main effect of measurement time (*F* (2, 354) = 1.16, *p* = 0.316, *η*^*2*^ = 0.002). That means no significant differences in sharing intention for accurate information between different intervention groups at different measurement times (Fig. 5).

## Discussion

The present study validated the effectiveness of a game-based inoculation in improving the perceived credibility and sharing intention of misinformation in China. The results support the efficacy of an intervention based on inoculation theory in countering misinformation. After the intervention, participants who accepted both game-based and graphic-based methods showed reduced perceived credibility and sharing intention of misinformation. This means that inoculation can activate people’s alertness to misinformation, allowing them to form more confident refutations of misinformation, thus reducing their acceptance of it and enabling them to understand and reject it better (van der Linden, 2022). In the 2-weeks follow-up, we found that both game-based and graphic-based inoculations had stable effects, similar to previous research findings (Maertens et al., 2021a, 2021b), demonstrating the value of inoculation in countering misinformation.

Additionally, the game-based intervention (*d* = − 0.89) demonstrated greater effectiveness in improving the perceived credibility of misinformation than the graphic-based intervention (*d* = − 0.45). This indicates that active inoculation had a better effect than passive inoculation. From a cognitive perspective, a possible reason might be that game-based interventions can better utilize multisensory stimuli such as visual, auditory, and tactile cues to improve learners’ memory and comprehension of information (Petri & Gresse von Wangenheim, 2017).

For the graphic-based intervention, we found that the perceived credibility of misinformation was not significantly different from the control group at the post-test stage. However, a significant decrease in perceived credibility was observed specifically during the follow-up stage, indicating the presence of a sleeper effect associated with passive inoculation. That means passive inoculation may undergo a process of enhancement before decaying. This result is consistent with the view of McGuire's, the proposer of inoculation theory: To enable individuals to develop arguments in defense of their attitude, it was imperative to introduce a time gap between the inoculation treatment and the attack message. This delay facilitated the necessary cognitive processing and response generation (Banas & Rains, 2010). The sleeper effect has also been concerned in attitudes and persuasion change (Kumkale & Albarracín, 2004). However, the perceived credibility of game-based inoculation significantly decreased at both the post-test and follow-up stages, suggesting that active inoculation directly activates immunity in individuals, further demonstrating the advantage of active inoculation over passive inoculation.

This study further examined the effects of inoculation on the perceived credibility and sharing intention of accurate information. The results showed that neither the game-based nor graphic-based intervention affected the perceived credibility and sharing intention of accurate information, indicating that the inoculation intervention did not weaken susceptibility to all information.

In practical applications, the game-based intervention designed in this study has advantages for implementing measures related to misinformation. This online gaming intervention can be implemented in a larger population, and with an increasing number of internet users, the intervention method is more suitable for social media dissemination than traditional offline or online teaching modes in terms of intervention time and scope (van der Linden et al., 2021). Furthermore, this approach can make targeted changes as misinformation changes in form and characteristics and is more likely to attract the public to engage in multiple interventions to actively strengthen the effect.

This study has some limitations. First, regarding the measurement materials, the misinformation measurement tool used in this study lacks standardization, which could lead to difficulties in comparing the results across different studies (Maertens et al., 2021a, 2021b). Therefore, future research should attempt to develop standardized measurement tools from a cross-cultural perspective and expand beyond questionnaire measurements to include behavioral indicators, such as attention time to content and critical information. Second, this study focused on misinformation in images and text commonly found on social media. However, with the explosive growth of short video forms and the widespread use of AI tools like ChatGPT, the prevalence of deep fakes and mixed true–false information is increasing, making it even more challenging to differentiate them (Hwang et al., 2021), future research should also pay attention to different forms of misinformation. Finally, the present study only performed a single follow-up measurement and did not examine the decay trend of intervention durability. Just as the duration of vaccine efficacy determines the timing of revaccination, we need to verify the continuity of changes in effectiveness among subjects after vaccination (Maertens et al., 2021a, 2021b). Future studies should measure the vaccine decay rate in response to inoculation at a single point (Goel et al., 2021).

## Conclusion

We cannot correct all misinformation; therefore, it makes sense to help the public prevent the danger of misinformation. Therefore, we designed a game-based inoculation to help the public against misinformation. The results also show that inoculation interventions can effectively deal with misinformation and game-based inoculation is more effective for misinformation perceived credibility. All interventions remained stable after 2 weeks, with no countereffects on the perceived credibility and sharing intention of accurate information.

## Supplementary Information

**Additional file 1**. Intervention game screenshots.

**Additional file 2**. Measurement materials.

## Data Availability

The data set with potentially identifying information removed and all analysis scripts have been made publicly available via the Open Science Framework (OSF) and can be accessed at: https://osf.io/g5hmb/.
