# Supplementary figures and images for: Game-based inoculation versus graphic-based inoculation to combat misinformation: a randomized controlled trial
Source: Cogn Res Princ Implic. 2023 Jul 31;8:49. doi: 10.1186/s41235-023-00505-x (PMC10390387; doi:10.1186/s41235-023-00505-x)

**Fig. S1**

*Intervention game screenshots*


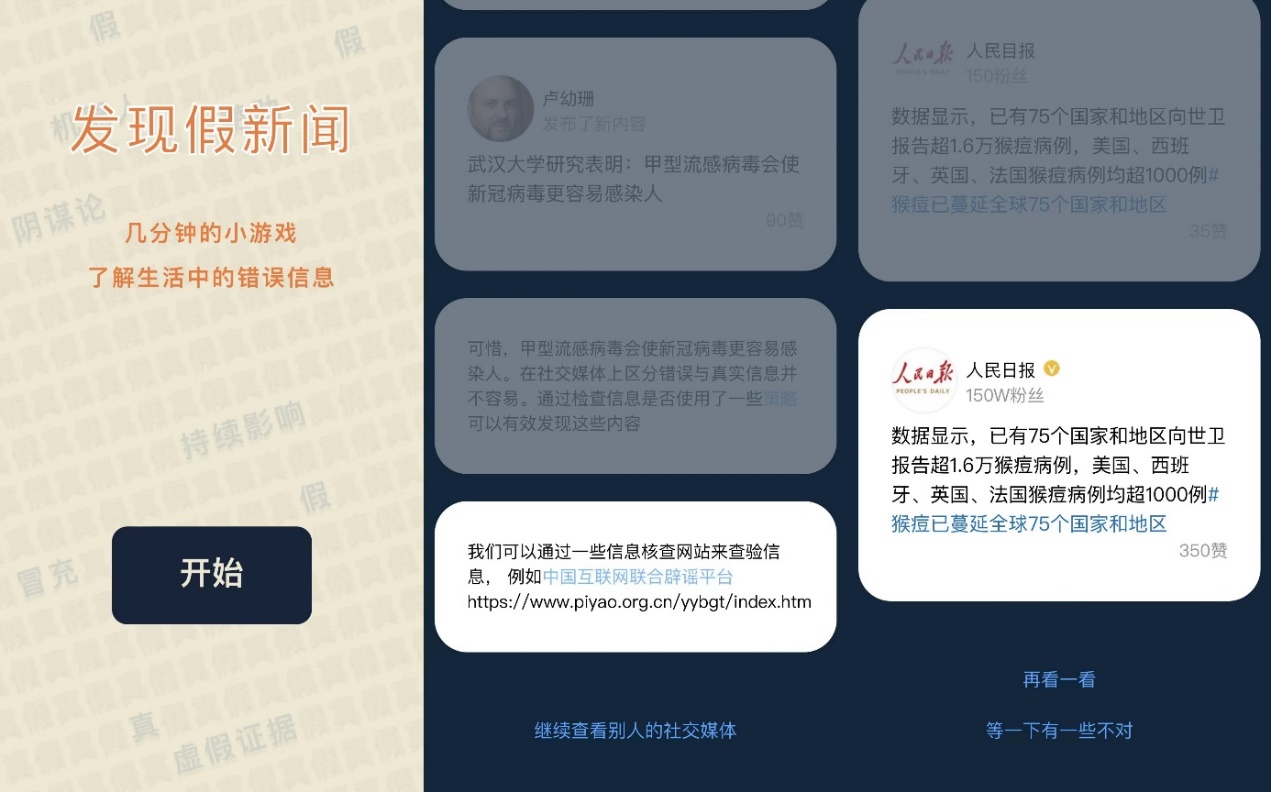

Supplement: Supplementary file 1 — Additional file 1. Intervention game screenshots. [file 41235_2023_505_MOESM1_ESM.docx]
