# Supplementary material for: Game-based inoculation versus graphic-based inoculation to combat misinformation: a randomized controlled trial
Source: Cogn Res Princ Implic. 2023 Jul 31;8:49. doi: 10.1186/s41235-023-00505-x (PMC10390387; doi:10.1186/s41235-023-00505-x)
Supplement: Supplementary file 2 — Additional file 2. Measurement materials. [file 41235_2023_505_MOESM2_ESM.docx]

# Measurement materials (English version)

## False news


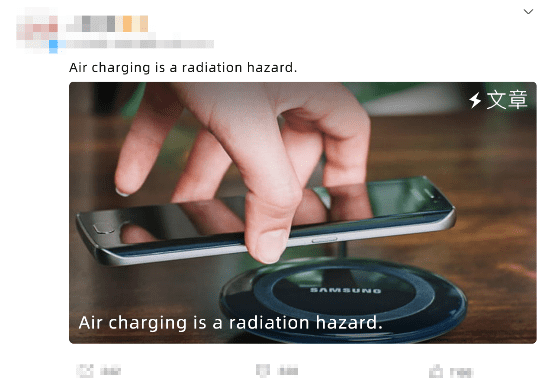


Question:

1. Is the news above accurate? (1=absolutely inaccurate, 5=absolutely accurate)

2. Is the news above accurate? (1=absolutely inaccurate, 5=absolutely accurate)


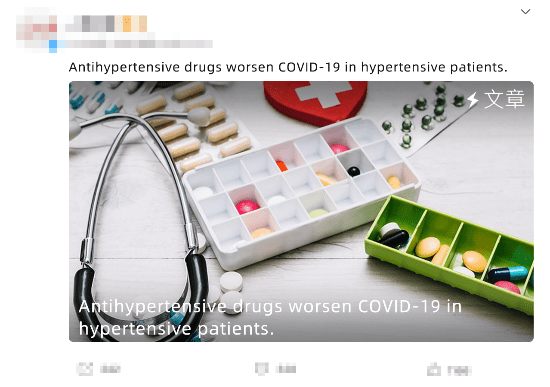


Question:

1. Is the news above accurate? (1=absolutely inaccurate, 5=absolutely accurate)

2. Is the news above accurate? (1=absolutely inaccurate, 5=absolutely accurate)


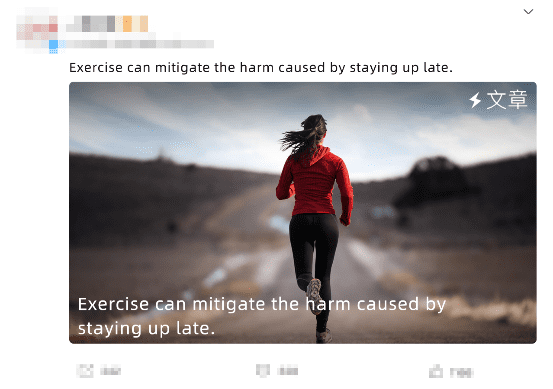
 Question:

1. Is the news above accurate? (1=absolutely inaccurate, 5=absolutely accurate)

2. Is the news above accurate? (1=absolutely inaccurate, 5=absolutely accurate)


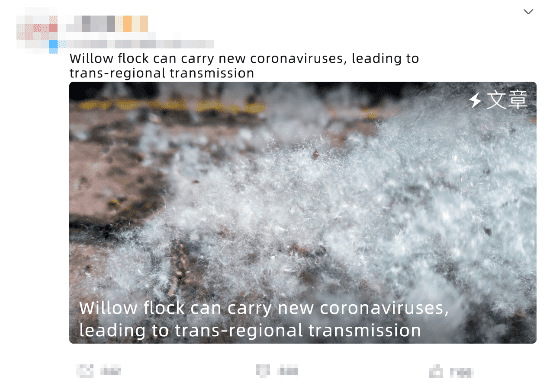
 Question:

1. Is the news above accurate? (1=absolutely inaccurate, 5=absolutely accurate)

2. Is the news above accurate? (1=absolutely inaccurate, 5=absolutely accurate)


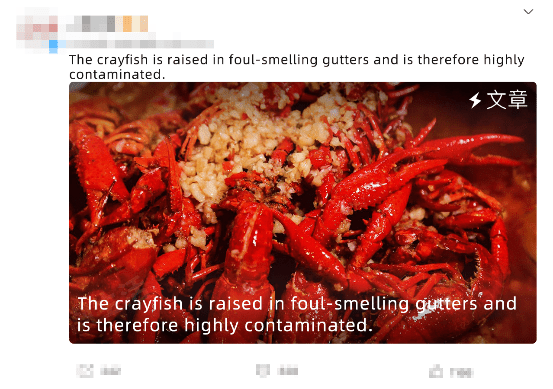
 Question:

1. Is the news above accurate? (1=absolutely inaccurate, 5=absolutely accurate)

2. Is the news above accurate? (1=absolutely inaccurate, 5=absolutely accurate)


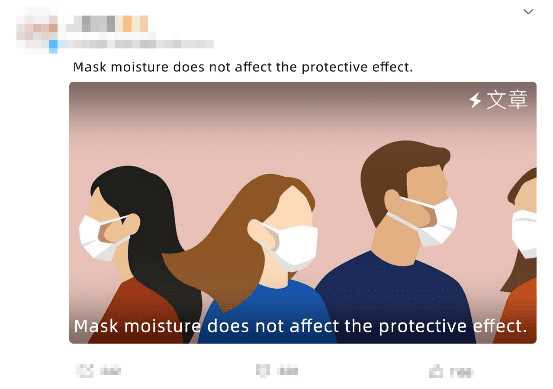
 Question:

1. Is the news above accurate? (1=absolutely inaccurate, 5=absolutely accurate)

2. Is the news above accurate? (1=absolutely inaccurate, 5=absolutely accurate)


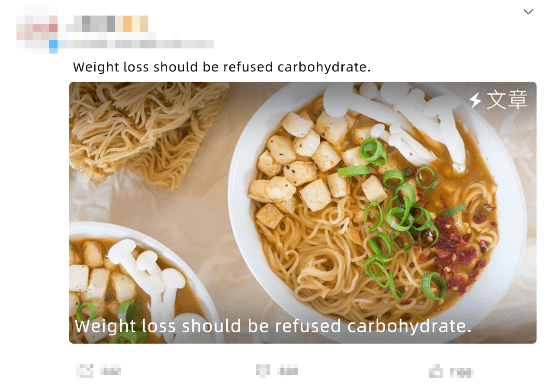
 Question:

1. Is the news above accurate? (1=absolutely inaccurate, 5=absolutely accurate)

2. Is the news above accurate? (1=absolutely inaccurate, 5=absolutely accurate)


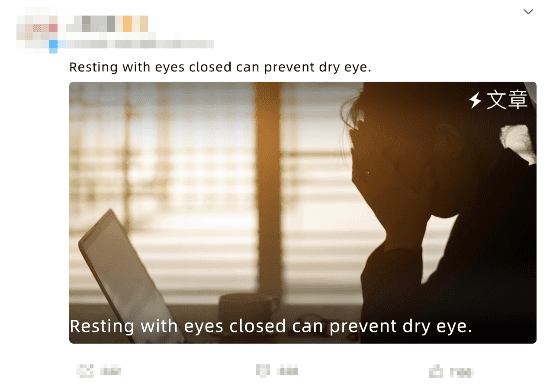
 Question:

1. Is the news above accurate? (1=absolutely inaccurate, 5=absolutely accurate)

2. Is the news above accurate? (1=absolutely inaccurate, 5=absolutely accurate)


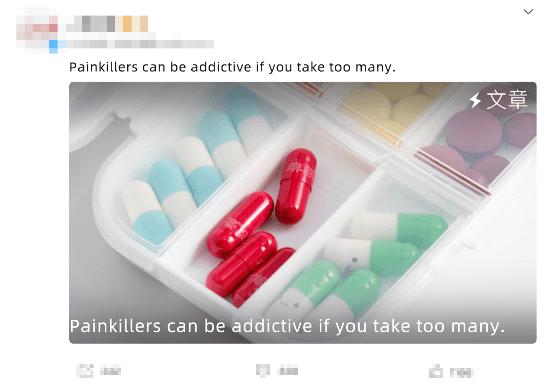
 Question:

1. Is the news above accurate? (1=absolutely inaccurate, 5=absolutely accurate)

2. Is the news above accurate? (1=absolutely inaccurate, 5=absolutely accurate)


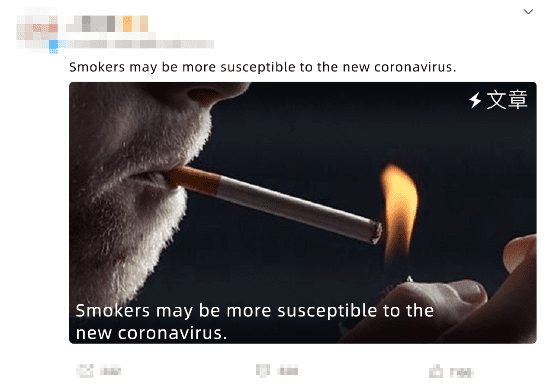
 Question:

1. Is the news above accurate? (1=absolutely inaccurate, 5=absolutely accurate)

2. Is the news above accurate? (1=absolutely inaccurate, 5=absolutely accurate)


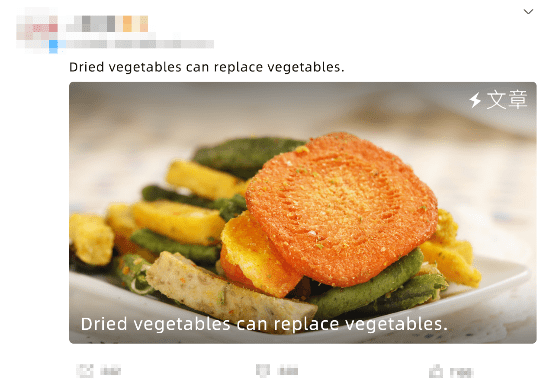
 Question:

1. Is the news above accurate? (1=absolutely inaccurate, 5=absolutely accurate)

2. Is the news above accurate? (1=absolutely inaccurate, 5=absolutely accurate)


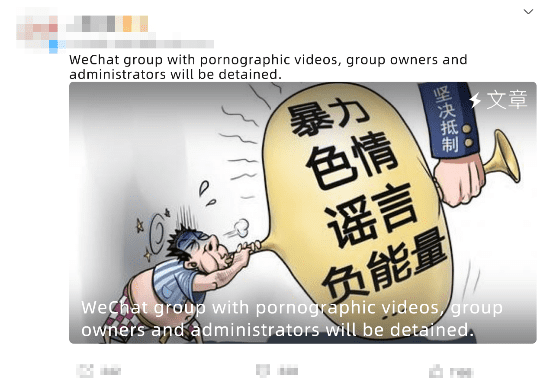
 Question:

1. Is the news above accurate? (1=absolutely inaccurate, 5=absolutely accurate)

2. Is the news above accurate? (1=absolutely inaccurate, 5=absolutely accurate)


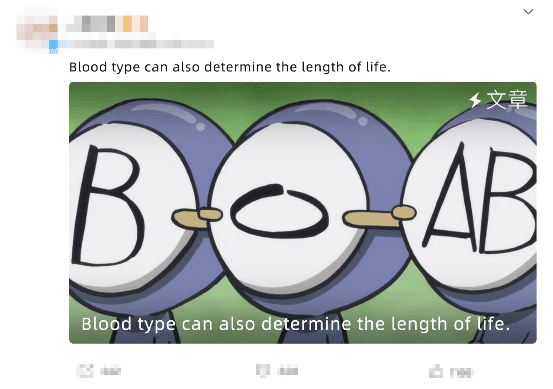
 Question:

1. Is the news above accurate? (1=absolutely inaccurate, 5=absolutely accurate)

2. Is the news above accurate? (1=absolutely inaccurate, 5=absolutely accurate)


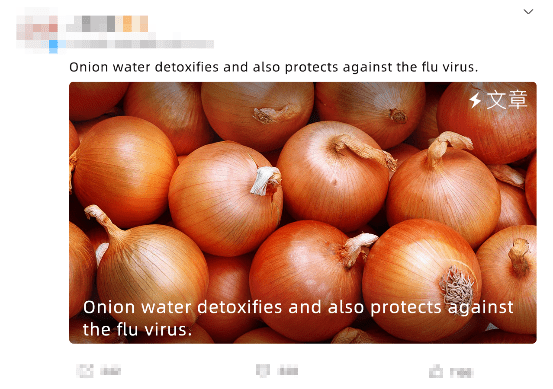
 Question:

1. Is the news above accurate? (1=absolutely inaccurate, 5=absolutely accurate)

2. Is the news above accurate? (1=absolutely inaccurate, 5=absolutely accurate)


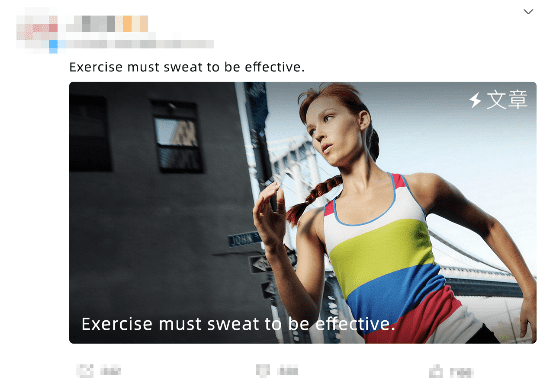
 Question:

1. Is the news above accurate? (1=absolutely inaccurate, 5=absolutely accurate)

2. Is the news above accurate? (1=absolutely inaccurate, 5=absolutely accurate)

## Real news


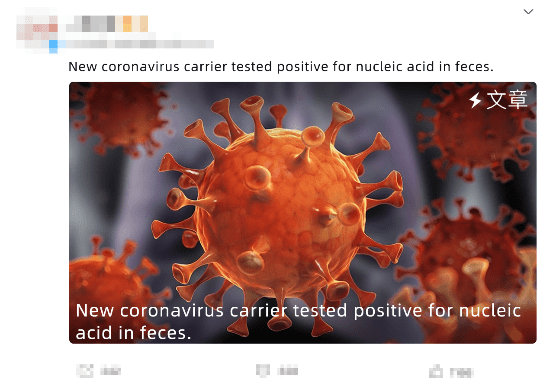
 Question:

1. Is the news above accurate? (1=absolutely inaccurate, 5=absolutely accurate)

2. Is the news above accurate? (1=absolutely inaccurate, 5=absolutely accurate)


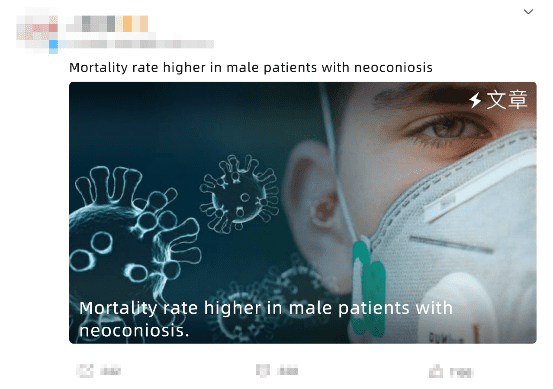
 Question:

1. Is the news above accurate? (1=absolutely inaccurate, 5=absolutely accurate)

2. Is the news above accurate? (1=absolutely inaccurate, 5=absolutely accurate)


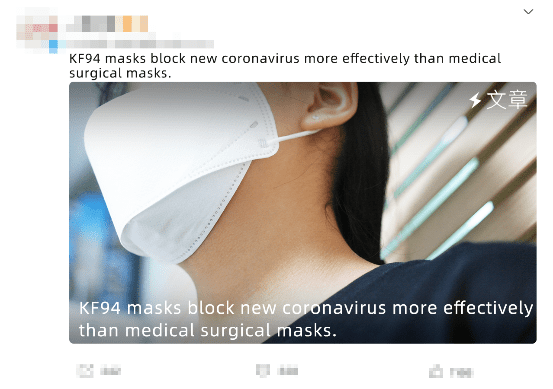
 Question:

1. Is the news above accurate? (1=absolutely inaccurate, 5=absolutely accurate)

2. Is the news above accurate? (1=absolutely inaccurate, 5=absolutely accurate)


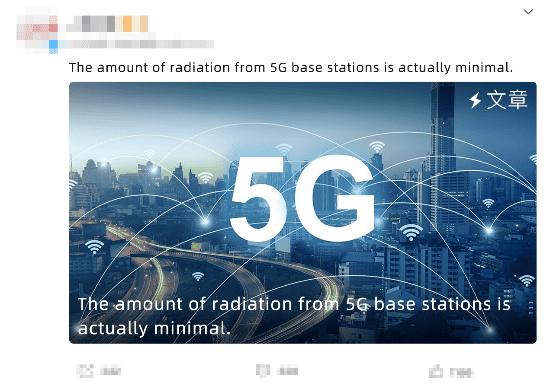
 Question:

1. Is the news above accurate? (1=absolutely inaccurate, 5=absolutely accurate)

2. Is the news above accurate? (1=absolutely inaccurate, 5=absolutely accurate)


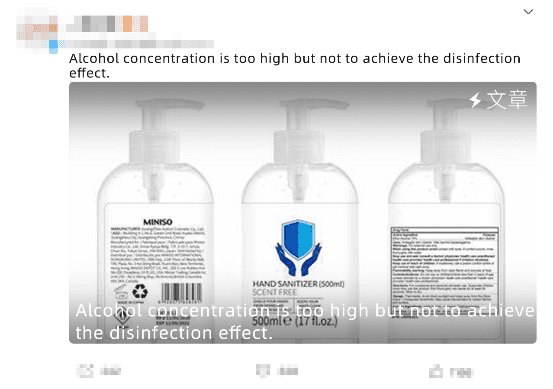
 Question:

1. Is the news above accurate? (1=absolutely inaccurate, 5=absolutely accurate)

2. Is the news above accurate? (1=absolutely inaccurate, 5=absolutely accurate)


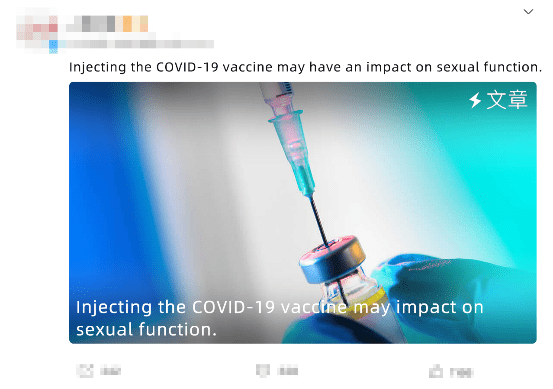
 Question:

1. Is the news above accurate? (1=absolutely inaccurate, 5=absolutely accurate)

2. Is the news above accurate? (1=absolutely inaccurate, 5=absolutely accurate)


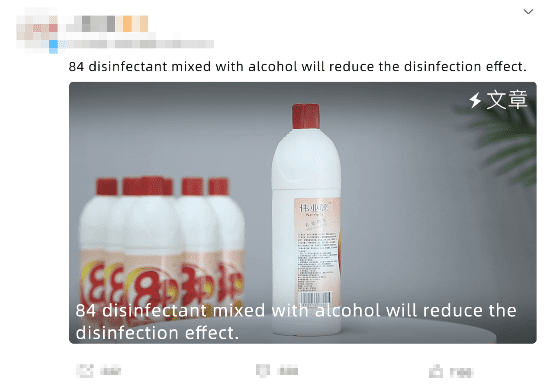
 Question:

1. Is the news above accurate? (1=absolutely inaccurate, 5=absolutely accurate)

2. Is the news above accurate? (1=absolutely inaccurate, 5=absolutely accurate)


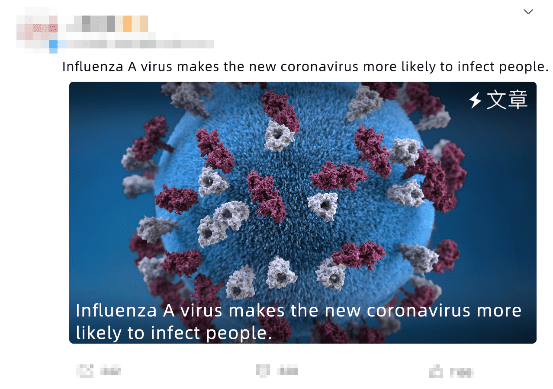
 Question:

1. Is the news above accurate? (1=absolutely inaccurate, 5=absolutely accurate)

2. Is the news above accurate? (1=absolutely inaccurate, 5=absolutely accurate)


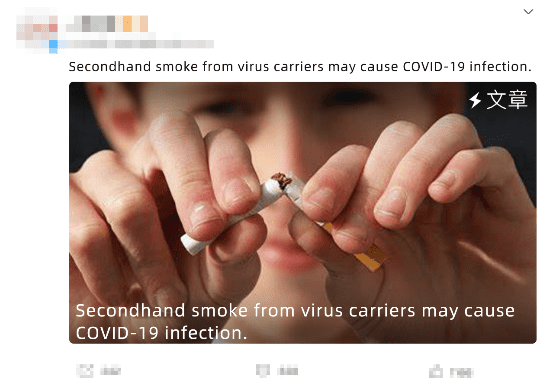
 Question:

1. Is the news above accurate? (1=absolutely inaccurate, 5=absolutely accurate)

2. Is the news above accurate? (1=absolutely inaccurate, 5=absolutely accurate)


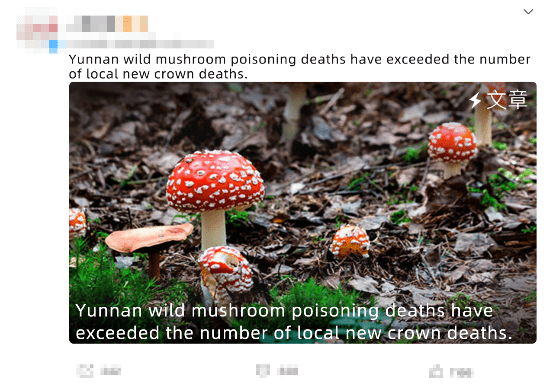
 Question:

1. Is the news above accurate? (1=absolutely inaccurate, 5=absolutely accurate)

2. Is the news above accurate? (1=absolutely inaccurate, 5=absolutely accurate)


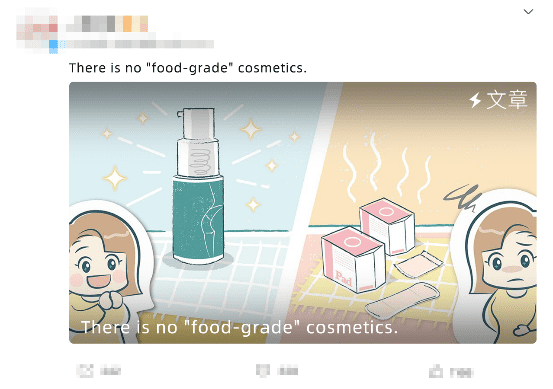
 Question:

1. Is the news above accurate? (1=absolutely inaccurate, 5=absolutely accurate)

2. Is the news above accurate? (1=absolutely inaccurate, 5=absolutely accurate)


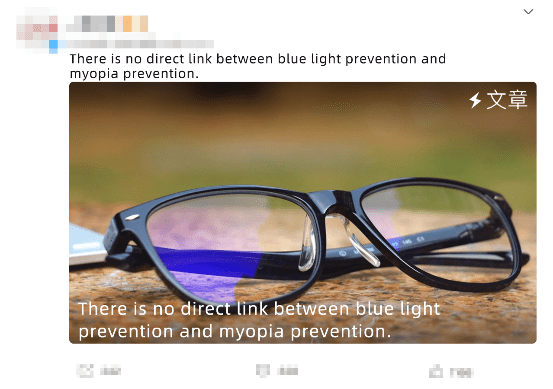
 Question:

1. Is the news above accurate? (1=absolutely inaccurate, 5=absolutely accurate)

2. Is the news above accurate? (1=absolutely inaccurate, 5=absolutely accurate)


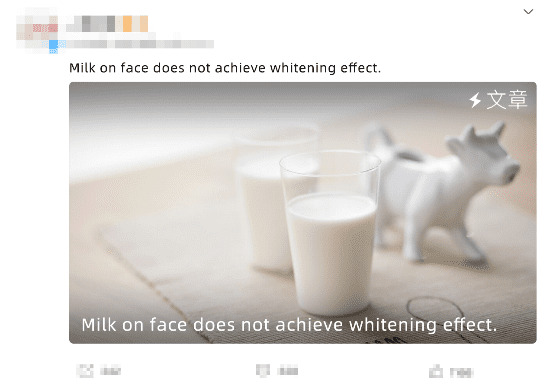
 Question:

1. Is the news above accurate? (1=absolutely inaccurate, 5=absolutely accurate)

2. Is the news above accurate? (1=absolutely inaccurate, 5=absolutely accurate)


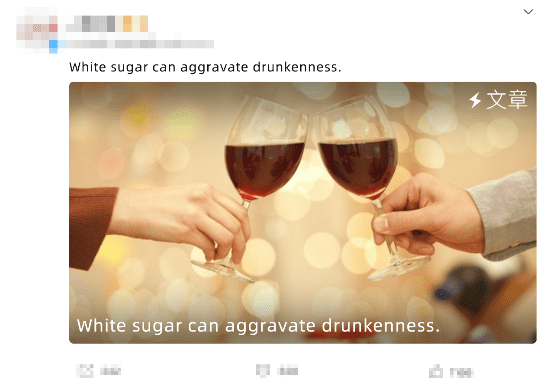
 Question:

1. Is the news above accurate? (1=absolutely inaccurate, 5=absolutely accurate)

2. Is the news above accurate? (1=absolutely inaccurate, 5=absolutely accurate)


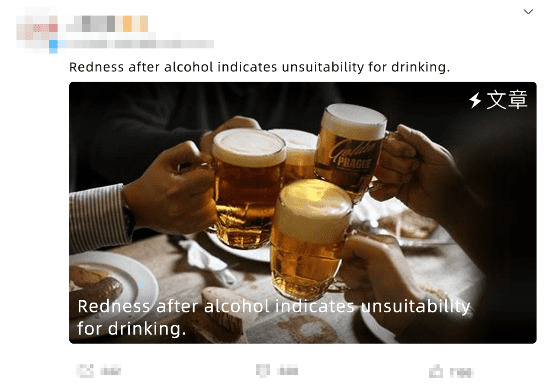


Question:

1. Is the news above accurate? (1=absolutely inaccurate, 5=absolutely accurate)

2. Is the news above accurate? (1=absolutely inaccurate, 5=absolutely accurate)

## Media literacy

I would follow the news using multiple media sources. (1 = Strongly disagree, 5 = Strongly agree)

I would contact with news organizations to show my reaction and tell my criticism. (1 = Strongly disagree, 5 = Strongly agree)

I would exchange information with my family or friends about the news I see in newspapers and on TV. (1 = Strongly disagree, 5 = Strongly agree)

I would caution people around me about the negative sides and negative effects of media. (1 = Strongly disagree, 5 = Strongly agree)

## Cognitive ability

The ages of Mark and Adam add up to 28 years total. Mark is 20 years older than Adam. How many years old is Adam?

If it takes 10 seconds for 10 printers to print out 10 pages of paper, how many seconds will it take 50 printers to print out 50 pages of paper?

On a loaf of bread, there is a patch of mold. Every day, the patch doubles in size. If it takes 40 days for the patch to cover the entire loaf of bread, how many days would it take for the patch to cover half of the loaf of bread?

If you’re running a race and you pass the person in second place, what place are you in?

Emily’s father has three daughters. The first two are named April and May. What is the third daughter’s name?

A farmer had 15 sheep and all but 8 died. How many are left?

# Measurement materials (Chinese version)

## False news


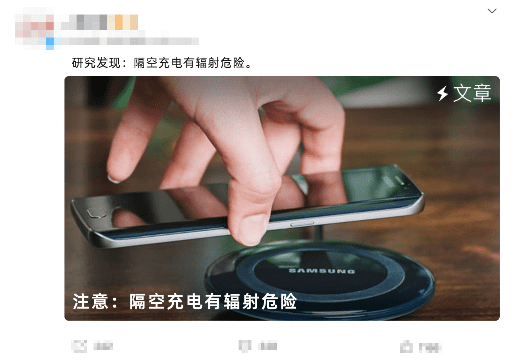


Question:

1. Is the news above accurate? (1=absolutely inaccurate, 5=absolutely accurate)

2. Is the news above accurate? (1=absolutely inaccurate, 5=absolutely accurate)


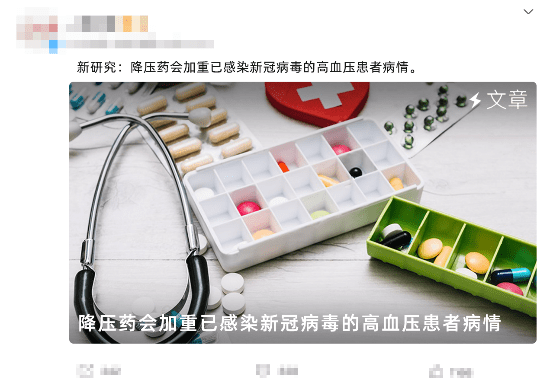
 Question:

1. Is the news above accurate? (1=absolutely inaccurate, 5=absolutely accurate)

2. Is the news above accurate? (1=absolutely inaccurate, 5=absolutely accurate)


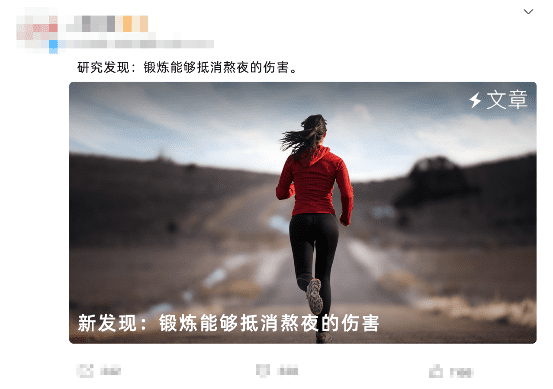
 Question:

1. Is the news above accurate? (1=absolutely inaccurate, 5=absolutely accurate)

2. Is the news above accurate? (1=absolutely inaccurate, 5=absolutely accurate)


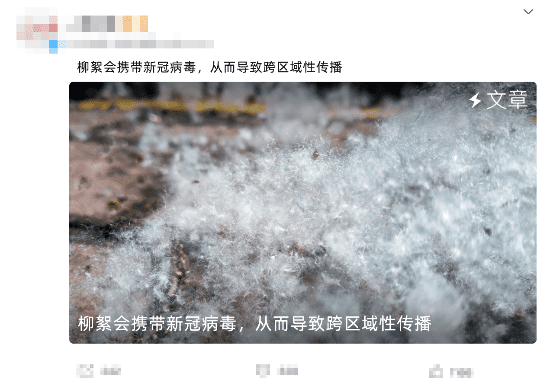
 Question:

1. Is the news above accurate? (1=absolutely inaccurate, 5=absolutely accurate)

2. Is the news above accurate? (1=absolutely inaccurate, 5=absolutely accurate)


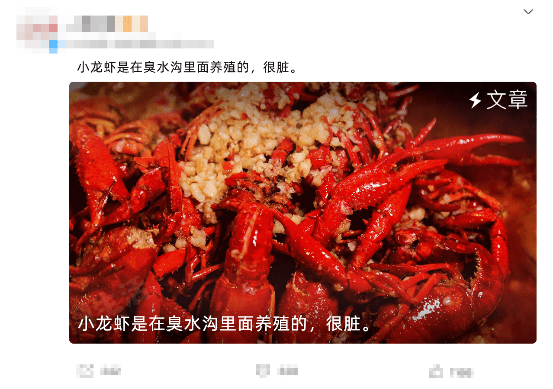
 Question:

1. Is the news above accurate? (1=absolutely inaccurate, 5=absolutely accurate)

2. Is the news above accurate? (1=absolutely inaccurate, 5=absolutely accurate)


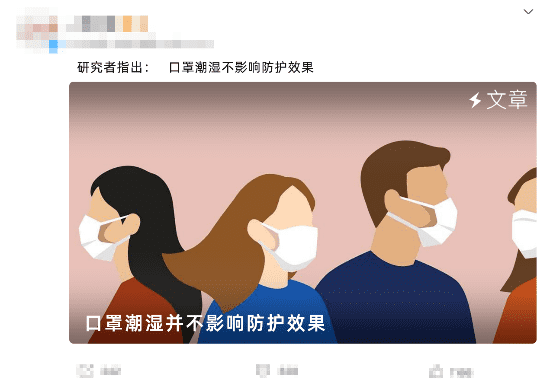
 Question:

1. Is the news above accurate? (1=absolutely inaccurate, 5=absolutely accurate)

2. Is the news above accurate? (1=absolutely inaccurate, 5=absolutely accurate)


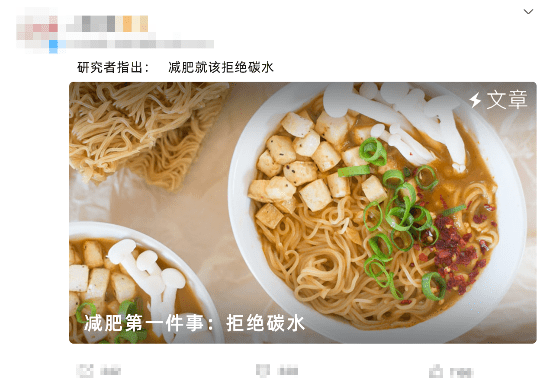
 Question:

1. Is the news above accurate? (1=absolutely inaccurate, 5=absolutely accurate)

2. Is the news above accurate? (1=absolutely inaccurate, 5=absolutely accurate)


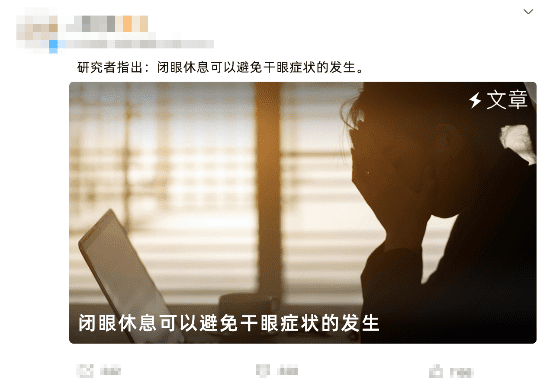
 Question:

1. Is the news above accurate? (1=absolutely inaccurate, 5=absolutely accurate)

2. Is the news above accurate? (1=absolutely inaccurate, 5=absolutely accurate)


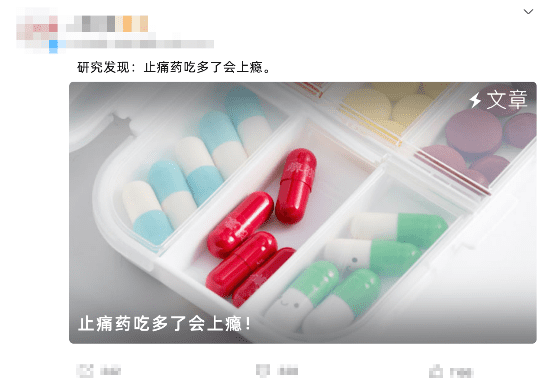
 Question:

1. Is the news above accurate? (1=absolutely inaccurate, 5=absolutely accurate)

2. Is the news above accurate? (1=absolutely inaccurate, 5=absolutely accurate)


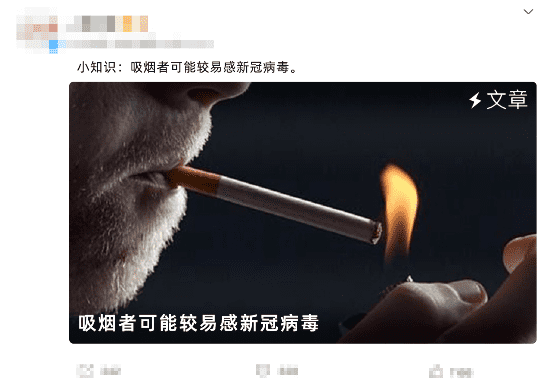
 Question:

1. Is the news above accurate? (1=absolutely inaccurate, 5=absolutely accurate)

2. Is the news above accurate? (1=absolutely inaccurate, 5=absolutely accurate)


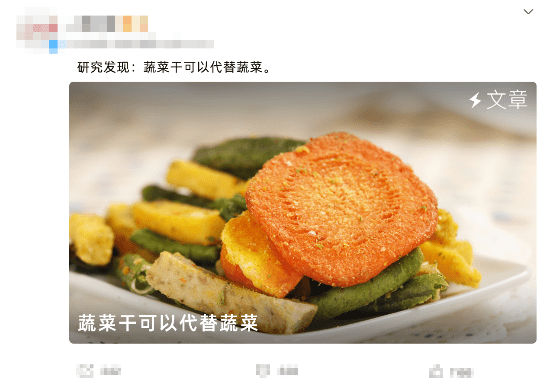
 Question:

1. Is the news above accurate? (1=absolutely inaccurate, 5=absolutely accurate)

2. Is the news above accurate? (1=absolutely inaccurate, 5=absolutely accurate)


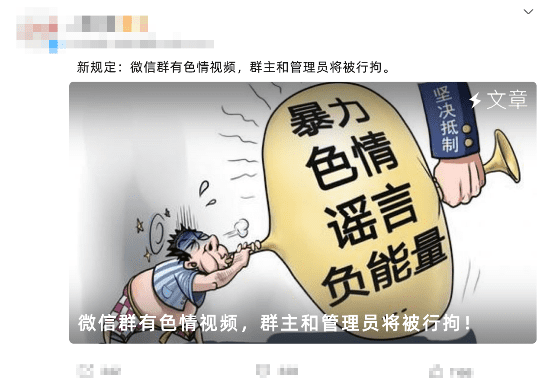
 Question:

1. Is the news above accurate? (1=absolutely inaccurate, 5=absolutely accurate)

2. Is the news above accurate? (1=absolutely inaccurate, 5=absolutely accurate)


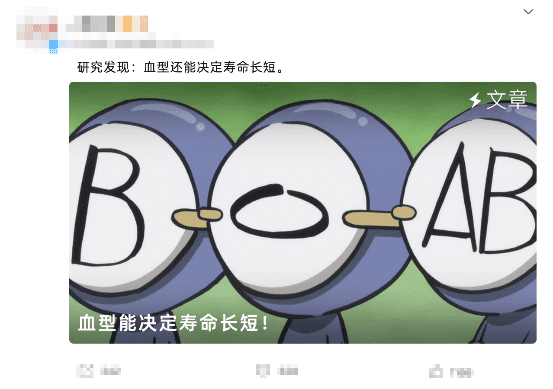
 Question:

1. Is the news above accurate? (1=absolutely inaccurate, 5=absolutely accurate)

2. Is the news above accurate? (1=absolutely inaccurate, 5=absolutely accurate)


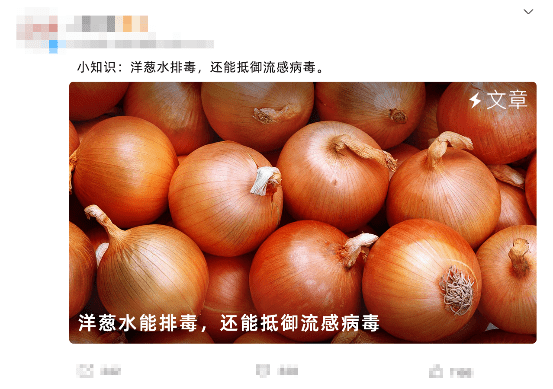
 Question:

1. Is the news above accurate? (1=absolutely inaccurate, 5=absolutely accurate)

2. Is the news above accurate? (1=absolutely inaccurate, 5=absolutely accurate)


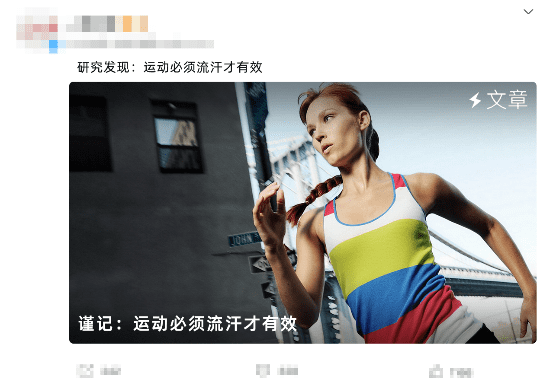
 Question:

1. Is the news above accurate? (1=absolutely inaccurate, 5=absolutely accurate)

2. Is the news above accurate? (1=absolutely inaccurate, 5=absolutely accurate)

## Real news


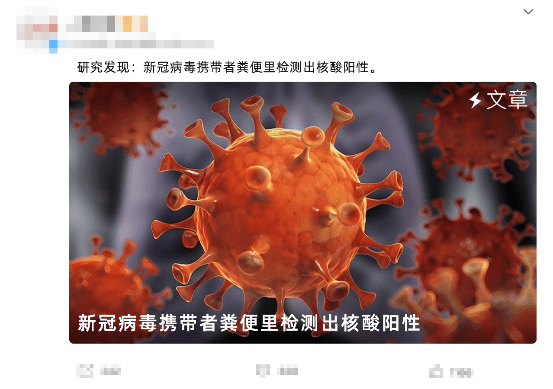
 Question:

1. Is the news above accurate? (1=absolutely inaccurate, 5=absolutely accurate)

2. Is the news above accurate? (1=absolutely inaccurate, 5=absolutely accurate)


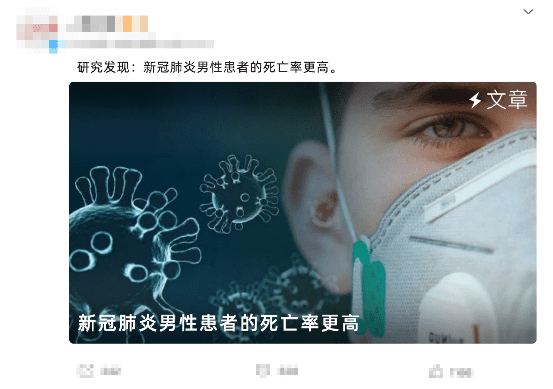
 Question:

1. Is the news above accurate? (1=absolutely inaccurate, 5=absolutely accurate)

2. Is the news above accurate? (1=absolutely inaccurate, 5=absolutely accurate)


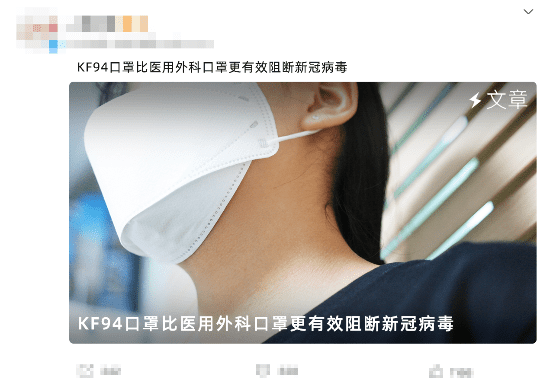
 Question:

1. Is the news above accurate? (1=absolutely inaccurate, 5=absolutely accurate)

2. Is the news above accurate? (1=absolutely inaccurate, 5=absolutely accurate)


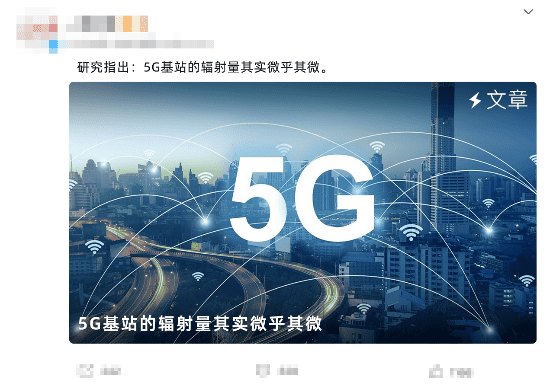
 Question:

1. Is the news above accurate? (1=absolutely inaccurate, 5=absolutely accurate)

2. Is the news above accurate? (1=absolutely inaccurate, 5=absolutely accurate)


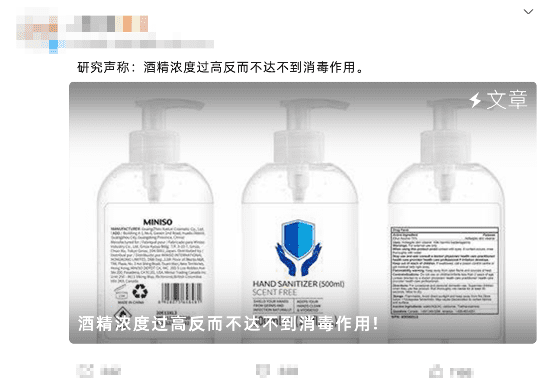
 Question:

1. Is the news above accurate? (1=absolutely inaccurate, 5=absolutely accurate)

2. Is the news above accurate? (1=absolutely inaccurate, 5=absolutely accurate)


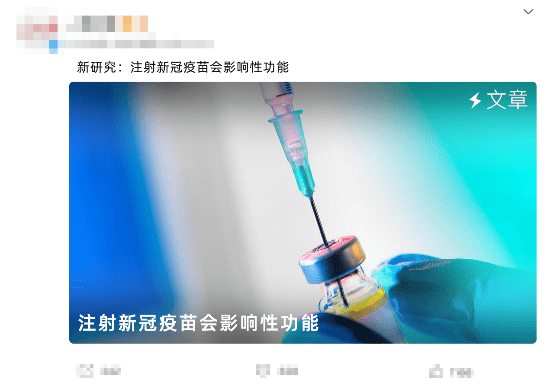
 Question:

1. Is the news above accurate? (1=absolutely inaccurate, 5=absolutely accurate)

2. Is the news above accurate? (1=absolutely inaccurate, 5=absolutely accurate)


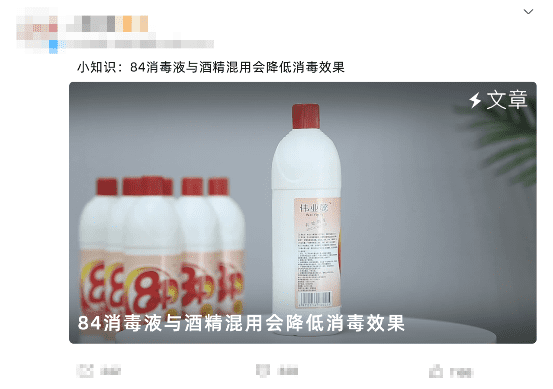
 Question:

1. Is the news above accurate? (1=absolutely inaccurate, 5=absolutely accurate)

2. Is the news above accurate? (1=absolutely inaccurate, 5=absolutely accurate)


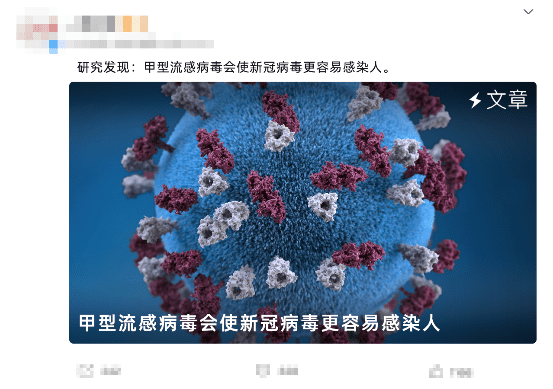
 Question:

1. Is the news above accurate? (1=absolutely inaccurate, 5=absolutely accurate)

2. Is the news above accurate? (1=absolutely inaccurate, 5=absolutely accurate)


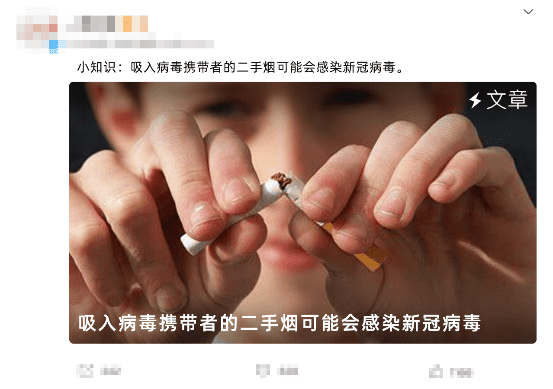
 Question:

1. Is the news above accurate? (1=absolutely inaccurate, 5=absolutely accurate)

2. Is the news above accurate? (1=absolutely inaccurate, 5=absolutely accurate)


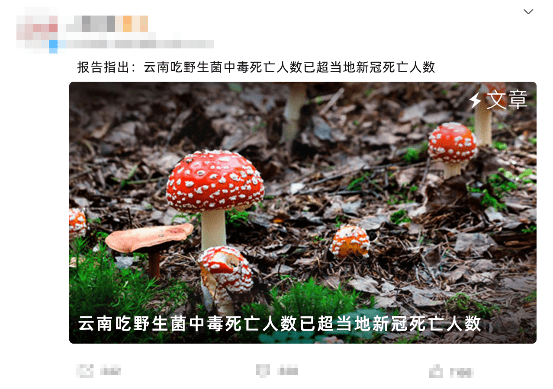
 Question:

1. Is the news above accurate? (1=absolutely inaccurate, 5=absolutely accurate)

2. Is the news above accurate? (1=absolutely inaccurate, 5=absolutely accurate)


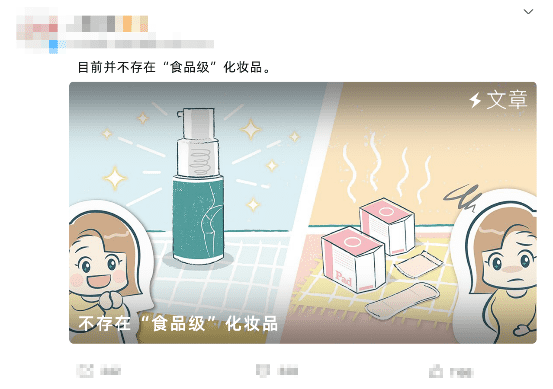
 Question:

1. Is the news above accurate? (1=absolutely inaccurate, 5=absolutely accurate)

2. Is the news above accurate? (1=absolutely inaccurate, 5=absolutely accurate)


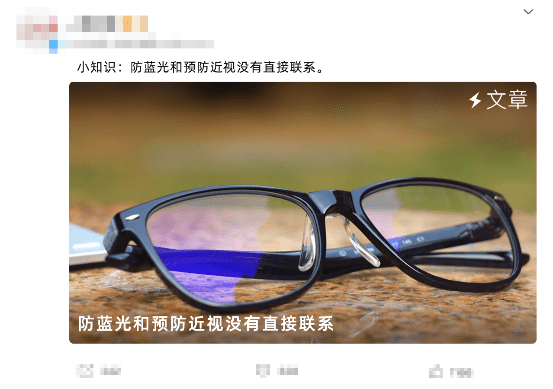
 Question:

1. Is the news above accurate? (1=absolutely inaccurate, 5=absolutely accurate)

2. Is the news above accurate? (1=absolutely inaccurate, 5=absolutely accurate)


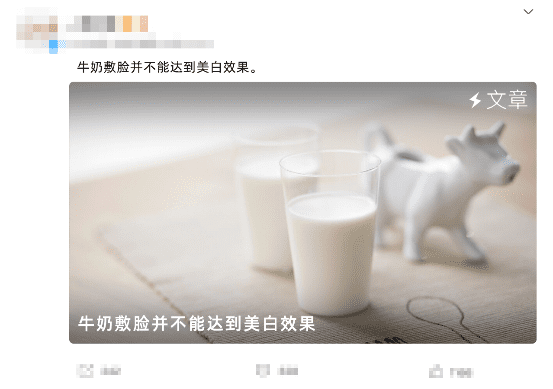
 Question:

1. Is the news above accurate? (1=absolutely inaccurate, 5=absolutely accurate)

2. Is the news above accurate? (1=absolutely inaccurate, 5=absolutely accurate)


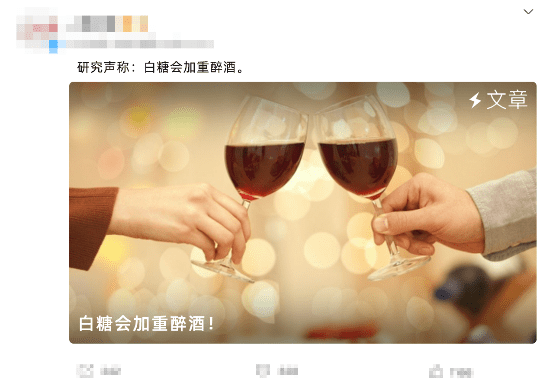
 Question:

1. Is the news above accurate? (1=absolutely inaccurate, 5=absolutely accurate)

2. Is the news above accurate? (1=absolutely inaccurate, 5=absolutely accurate)


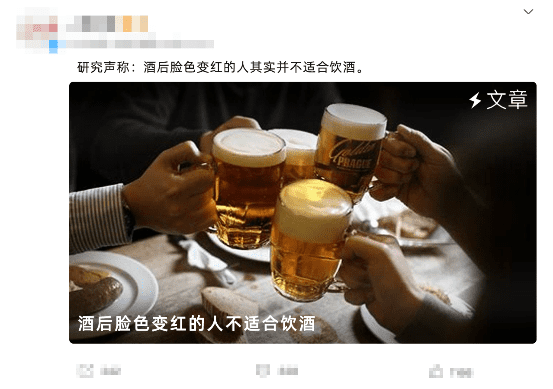


Question:

1. Is the news above accurate? (1=absolutely inaccurate, 5=absolutely accurate)

2. Is the news above accurate? (1=absolutely inaccurate, 5=absolutely accurate)

## Media literacy

I would follow the news using multiple media sources. (1 = Strongly disagree, 5 = Strongly agree)

I would contact with news organizations to show my reaction and tell my criticism. (1 = Strongly disagree, 5 = Strongly agree)

I would exchange information with my family or friends about the news I see in newspapers and on TV. (1 = Strongly disagree, 5 = Strongly agree)

I would caution people around me about the negative sides and negative effects of media. (1 = Strongly disagree, 5 = Strongly agree)

## Cognitive ability

The ages of Mark and Adam add up to 28 years total. Mark is 20 years older than Adam. How many years old is Adam?

If it takes 10 seconds for 10 printers to print out 10 pages of paper, how many seconds will it take 50 printers to print out 50 pages of paper?

On a loaf of bread, there is a patch of mold. Every day, the patch doubles in size. If it takes 40 days for the patch to cover the entire loaf of bread, how many days would it take for the patch to cover half of the loaf of bread?

If you’re running a race and you pass the person in second place, what place are you in?

Emily’s father has three daughters. The first two are named April and May. What is the third daughter’s name?

A farmer had 15 sheep and all but 8 died. How many are left?
